# Patient-derived prostate cancer organoids: model development, therapeutic applications, and challenges for precision oncology

**DOI:** 10.3389/fonc.2026.1939330

**Published:** 2026-09-18

**Authors:** Jingzhen Zhu, Sheng Wang, Furong Du, Sihai Shao

**Affiliations:** 1 Department of Urology, Urologic Surgery Center, Xinqiao Hospital, Third Military Medical University (Army Medical University), Chongqing, China; 2 Department of Urology, Changzhou Hospital of Traditional Chinese Medicine, Changzhou Affiliated Hospital of Nanjing University of Chinese Medicine, Changzhou, Jiangsu, China; 3 Department of Medicine, Kingbio Medical Co., Ltd., Chongqing, China; 4 Department of Urology, First Affiliated Hospital of Huzhou Normal University, Huzhou, China

**Keywords:** drug resistance, patient-derived organoids, precision medicine, prostate cancer, tumor heterogeneity

## Abstract

Prostate cancer ranks among the most prevalent male malignancies worldwide, with castration-resistant prostate cancer (CRPC) and drug resistance representing major clinical challenges. Conventional preclinical models fail to faithfully recapitulate tumor heterogeneity and the tumor microenvironment (TME). Patient-derived prostate cancer organoids (PCOs) represent a promising 3D model that largely preserves the genomic, phenotypic, and functional signatures of primary tumors. Building on prior studies that mostly focus on fragmented analyses and lack in-depth systematic integration, this narrative review attempts to establish a refined multidimensional research framework centered on “model optimization-mechanism exploration-clinical translation”. Notably, it underscores the unique advantages of engineered PCOs and heterotypic co-culture organoid systems in resolving key clinical bottlenecks, particularly lineage transformation-driven therapeutic resistance and patient-specific TME heterogeneity. This study systematically elaborates the establishment methodologies of PCOs, critically evaluates the strengths, limitations, and iterative optimization strategies of diverse culture systems, and summarizes the core applications of PCOs in exploring tumor cellular origin, capturing tumor heterogeneity, modeling TME interactions, screening drug sensitivity, investigating resistance mechanisms, and advancing precision medicine for prostate cancer. Although PCO-based platforms still possess inherent limitations, including low establishment efficiency, rigorous culture requirements, and incomplete TME reconstruction, they harbor substantial translational potential. Furthermore, this review proposes targeted optimization strategies based on bioengineering and synthetic biomaterials, prospects the clinical translation of standardized PCO systems, and discusses existing research contradictions and unaddressed gaps in this field, aiming to provide a comprehensive, critical and novel theoretical reference for further basic research and clinical application of PCOs in prostate cancer.

## 1 Introduction

Prostate cancer ranks as the second most prevalent malignancy in men worldwide, following only lung cancer. In 2022, there were an estimated 1 466–680 newly diagnosed cases of prostate cancer and 396–792 deaths (1). Most prostate malignancies are luminal-type adenocarcinomas that express the transcription factor androgen receptor (AR) and exhibit marked reliance on AR signaling cascades (2). For localized prostate cancer, surgical resection or radiotherapy yields favorable treatment outcomes, but approximately 20% of patients eventually develop fatal recurrent disease (3). Clinical management of recurrent prostate cancer relies on AR signaling inhibition; however, this approach nearly inevitably drives progression to metastatic castration-resistant prostate cancer (CRPC) (4). Although CRPC usually preserves an AR-dependent adenocarcinoma phenotype, approximately 20% of cases exhibit phenotypic plasticity, undergoing differentiation into alternative cell lineages including the neuroendocrine phenotype (5, 6). Over the past decade, substantial advances in delineating the genetic determinants of prostate cancer have been achieved, and significant genetic heterogeneity in CRPC has also been demonstrated (7–9). Building on this genetic framework, systematic investigations into the cellular and molecular features of prostate cancer are pivotal for elucidating the mechanisms underlying tumor initiation, disease progression, and treatment resistance, as well as for developing novel treatment strategies. To advance such mechanistic and translational research effectively, robust preclinical models with high biological fidelity and experimental tractability are indispensable.

Numerous preclinical models have been utilized to propel prostate cancer research. In most studies, immortalized cell lines, either cultured in two-dimensional (2D) monolayers or xenografted into immunocompromised murine models, are usually adopted. Despite the convenience and widespread availability of these cell lines, their scarcity and the phenotypic drift induced by the prolonged *in vitro* culture make them a poor surrogate for primary tumor tissue (10). Moreover, a key shortcoming of cell line models is their inability to capture the diverse dimensions of intratumoral and intertumoral heterogeneity (11). Patient-derived xenografts (PDXs) are considered to be a superior preclinical model for prostate cancer due to their ability to recapitulate the phenotypic features of the parental tumor (12). Despite their unique advantage of incorporating a tumor-associated microenvironment, PDX models are constrained by their requirement for immunocompromised murine hosts and lack of feasibility for high-throughput therapeutic evaluation. In addition, it usually takes several months to generate PDX models for drug screening, which is not only time-consuming but also costly (13). Patient-derived tumor organoids (PDTOs) are three-dimensional (3D) *in vitro* cultures derived from patient tumor tissues or stem cells. By bridging the conventional gaps between patient-derived cancer cells and PDX models, PDTOs can largely simulate the pathophysiological features of spontaneous tumorigenesis and metastasis. Meanwhile, they have driven the development of patient-specific drug screening platforms, and personalized therapeutic strategies, as well as the identification of prognostic biomarkers and drug resistance mechanisms (14). Prostate cancer organoids (PCOs) are established from malignant prostate tumors and frequently benchmarked against normal prostate organoids in mechanistic and translational studies. They can effectively model AR-dependent and neuroendocrine phenotypes, support high-throughput drug sensitivity testing for precision medicine, and help clarify treatment resistance mechanisms, which facilitates the selection of individualized therapeutic strategies for patients with multiple treatment options and promotes translational research in prostate cancer (15, 16). Notably, normal prostate organoids are 3D epithelial culture models derived from benign human prostate tissues or healthy prostate epithelial cells. They recapitulate the physiological epithelial architecture, cellular composition and biological properties of native prostate tissue, serving as essential controls for identifying tumor-specific malignant phenotypes.

Building on prior studies that mostly focus on fragmented analyses without in-depth integrated synthesis this narrative review attempts to establish a refined multidimensional research framework centered on “model optimization-mechanism exploration-clinical translation”. It elaborates the establishment methodologies of PCOs, critically evaluates the strengths, limitations and iterative optimization strategies of diverse culture systems, and summarizes the core applications of PCOs in exploring tumor cellular origin, capturing tumor heterogeneity, modeling tumor microenvironment (TME) interactions, screening drug sensitivity, investigating drug-resistant mechanisms, and advancing precision medicine for prostate cancer (**Figure 1**). Meanwhile, this review proposes bioengineering and biomaterial-based optimization approaches, and highlights ongoing controversies and unaddressed research gaps, aiming to deliver a comprehensive, updated theoretical framework to support fundamental and translational research utilizing PCOs.

**Figure 1:**
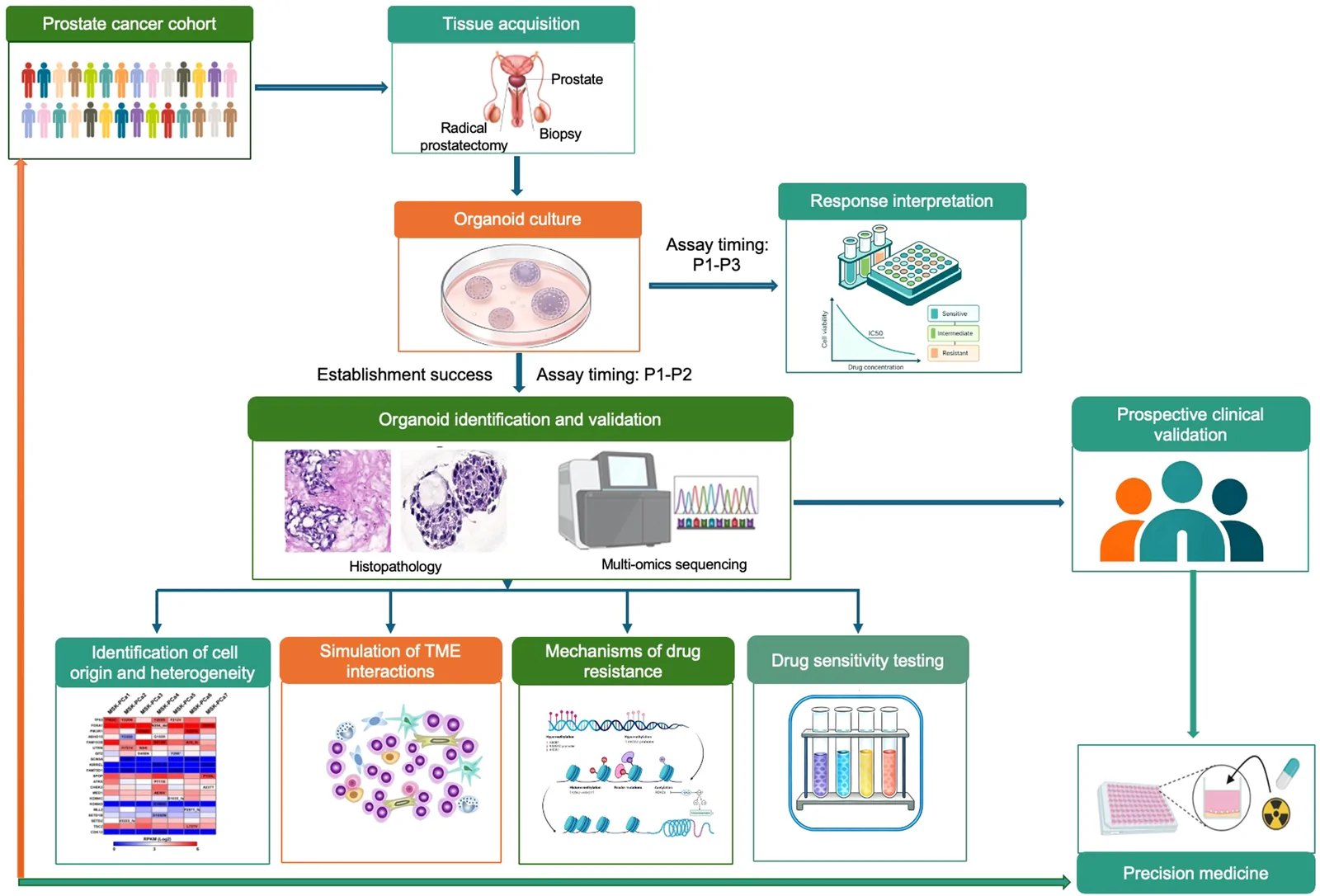
Contribution of patient-derived prostate cancer organoids to accelerating clinical translation. Prostate tumor tissues from biopsies or radical prostatectomy are used to establish prostate cancer organoids. They are validated at histological and multi-omics levels at early passages (P1-P2). Validated organoids enable diverse pre-clinical studies, including characterization of cellular origin and tumor heterogeneity, simulation of tumor microenvironment interactions, investigation of drug-resistance mechanisms, and drug-sensitivity testing. Drug response is assessed at passages P1-P3. Findings from organoid-based studies further support prospective clinical validation and ultimately guide precision medicine practice for prostate cancer patients.

## 2 Literature search strategies

Unlike systematic reviews with standardized retrieval and screening procedures, the present work is a narrative review focusing on thematic integration, comprehensive evidence discussion and prospective analysis, rather than rigid systematic evidence synthesis. Relevant literature was extensively retrieved and manually screened to summarize current research progress, controversies and research gaps in the field of PCOs. Publications were collected from PubMed, Web of Science, and Embase up to March 2026 using the core keywords: “prostate cancer”, “patient-derived organoids”, “tumor heterogeneity”, “drug resistance”, “tumor microenvironment”, “precision medicine”. Inclusion criteria: (1) original basic and translational studies reporting PCO establishment, optimization, mechanistic exploration and clinical application; (2) systematic reviews and meta-analyses related to PCO translational research; (3) high-quality studies on engineered organoids and TME simulation models. Exclusion criteria: (1) case reports, letters, conference abstracts without complete data; (2) repetitive published studies, non-English literatures, and studies focusing solely on other tumor organoids without prostate cancer core data.

Most existing reviews simply list discrete experimental data without multidimensional thematic organization. Herein, we categorize relevant literature into five core themes: organoid model optimization, intratumoral heterogeneity verification, TME interactions, drug resistance mechanisms, and clinical translation. By cross-analyzing independent studies, we consolidate consistent findings, clarify conflicting observations, and provide a holistic critical perspective beyond conventional chronological summaries.

## 3 Laboratory methodologies utilized to generate patient-derived PCOs

The early advancement of prostate 3D culture technology was achieved through the establishment of spheroid culture models derived from mouse prostate tissues. This experimental approach entailed the *in vitro* culture of intact or dissociated mouse prostate epithelial tissues in commercially available serum-free medium supplemented with a 3D extracellular matrix (ECM), which ultimately promoted the formation of spheroids possessing the capacities for self-renewal and self-organized differentiation (17, 18). Matrigel has long been recognized as a core component underpinning the initial success of organoid culture systems. Leveraging these early methodological strategies, researchers were able to establish 3D prostate culture models by sustaining the proliferation of luminal cells and subsequently supplementing the culture medium with androgens, such as dihydrotestosterone. Although this approach enabled partial differentiation of luminal cells into intermediate phenotypes with AR expression, the established spheroid models failed to recapitulate the characteristic secretory phenotype of the prostate luminal epithelium (19). Subsequently, organoid models from normal prostate tissues that comprised both basal and luminal cells were successfully established (18, 20), and PCOs were generated from biopsy samples of metastatic prostate cancer and circulating prostate cancer cells (18). These 3D models including normal prostate organoids and PCOs (**Figure 2**) can serve as valuable tools to investigate the core molecular mechanisms that govern the initiation and/or progression of prostate cancer.

**Figure 2:**
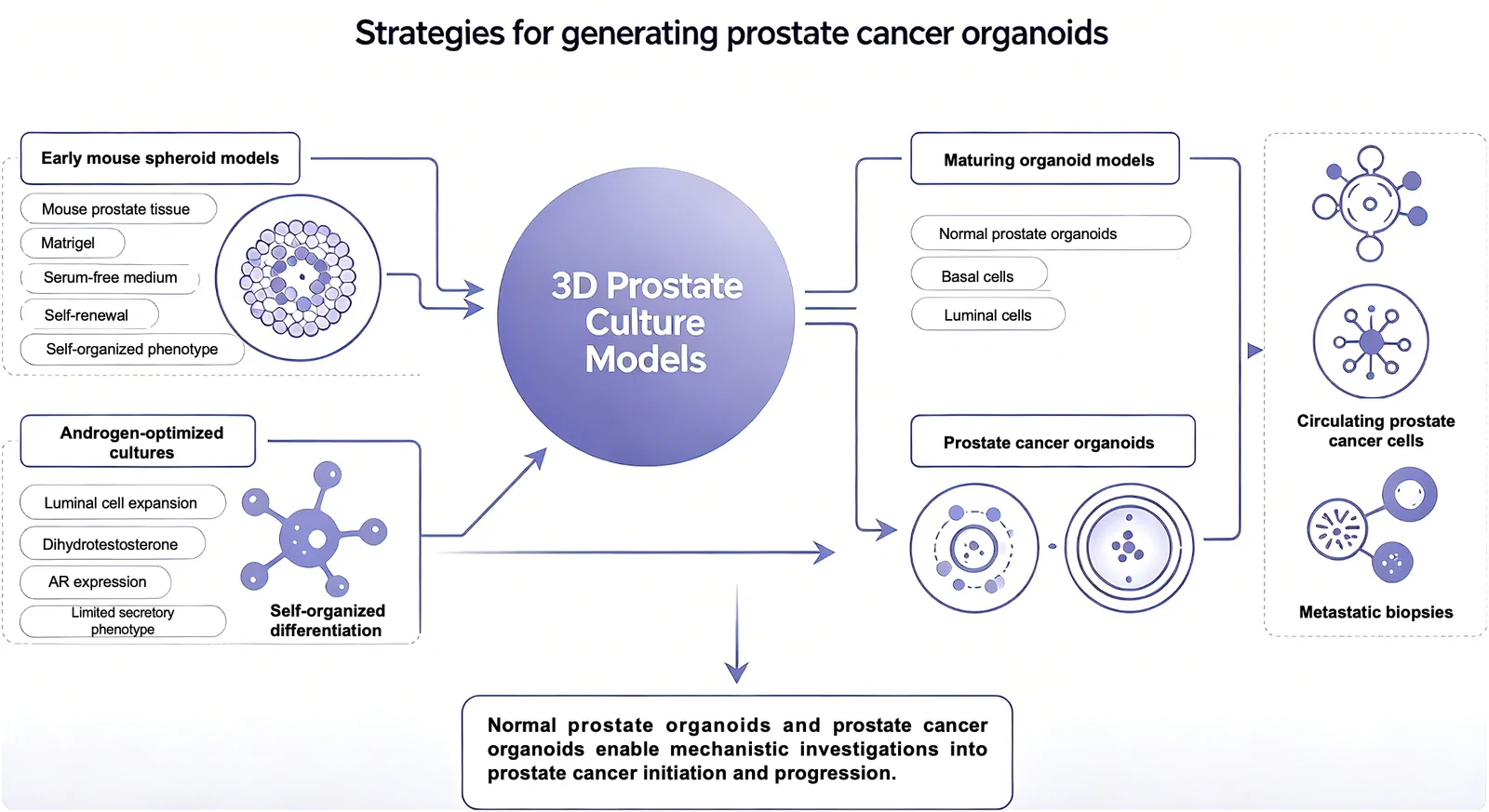
Generation of normal prostate organoids and prostate cancer organoids. Early mouse prostate spheroid cultures were generated using Matrigel-embedded epithelial cells in serum-free medium. Despite androgen-driven luminal proliferation and AR expression, these spheroids incompletely recapitulated luminal secretory features. Optimized culture protocols yield normal prostate organoids containing basal and luminal compartments. Importantly, prostate cancer organoids can be derived from metastatic biopsies and circulating tumor cells. Normal prostate organoids and prostate cancer organoids may both serve as valuable tools for unravelling mechanisms of prostate cancer onset and progression.

In the research field of patient-derived organoids, intestinal epithelial organoids from Lgr5-expressing stem cells were first generated, and long-term organoid culture was sustained by supplementing the medium with key proliferative factors, such as R-spondin1 and Noggin, and a Rho kinase inhibitor (21). Subsequent advancements involved the combination of the Rho kinase inhibitor Y-27632 with fibroblast feeder cells to induce conditional cell reprogramming (22). This approach facilitates the rapid and long-term expansion of tumor cells isolated from small biopsy samples, and has been applied to both early- and advanced-stage prostate cancer at differing efficiencies.

Regarding the establishment of PCOs, there are a variety of scaffolds and culture conditions (**Table 1**) (15, 20, 23–30). Matrigel has proven to be a dependable artificial basement membrane extract (BME), and has been used to generate PCOs in some studies. It is abundant in basement-membrane proteins, including laminin, collagen, entactin/nidogen and proteoglycans, which sustain the growth and differentiation of non-malignant and malignant prostate epithelial cells. However, it has notable drawbacks, including animal-derived origin, batch-to-batch inconsistency, and multiple undefined components that can impact organoid development. A microwell platform based on polydimethylsiloxane has also been utilized to culture prostate cells, yielding size-controlled microaggregates of these cells (31). Importantly, through a microwell flow device that generates in-well laminar flow around 3D tissues, the cell-line-based cultured 3D prostate models exhibit improved chemotherapeutic sensitivity and more stable transcriptional responses (32). Moreover, a novel hyaluronan-based 3D hydrogel system has been developed to improve the viability of bone-metastatic PDX tumor cells. When encapsulated within this hydrogel, PDX cells can be maintained in ex vivo culture, facilitating gene editing, drug screening, and mechanistic investigations of prostate cancer bone-tropic metastasis. In a previous study, the organoids displayed increased chemoresistance, partly attributable to heterogeneous chemical gradients and restricted drug penetration within 3D architectures, as seen in 3D cultures of other solid tumors (33).

**Table 1:** Methods for culturing and establishing prostate cancer organoids.

Sample source (patient-derived/PDX-derived)	Disease stage	3D scaffold & matrix concentrations	Basal medium (supplements)	Establishment success rate	Organoid characteristics	Ref No.
Patient-derived biopsy samples + LuCaP PDX-derived tumor tissues	Advanced prostate cancer	Matrigel; For 12-well plating: 20 μL cold culture medium containing cells mixed with 80 μL growth factor-reduced phenol-red-free Matrigel. For 48-well plating: cells resuspended directly in 20 μL Matrigel. For in-vivo organoid-derived xenograft injection: partially dissociated organoids mixed with Matrigel at 1:1 (v/v) ratio.	Advanced DMEM/F12 (EGF, Noggin, R-spondin 1, A83-01, DHT, FGF2, FGF10, PGE2, nicotinamide, NAC, SB202190 (p38i), B27; Y-27632 added during seeding only)	>50% for long-term PDX-derived organoid culture	Lineage markers and transcriptomic profiles are both preserved between PDXs and PCOs.	(15)
Patient-derived metastatic prostate cancer biopsy tissues + CRPC CTCs	Advanced/metastatic CRPC	Matrigel; NR	Advanced DMEM/F12 (EGF, Noggin, R-spondin 1, A83-01, DHT, FGF2, FGF10, PGE2, nicotinamide, NAC, SB202190 (p38i), B27; Y-27632 added during seeding only)	15%-20% for long-term culture; ~70% of soft tissue tumor biopsies and ~30% of bone biopsies for short-term culture	The long-term culture of PCOs from biopsies and CTCs were successful, and organoid lines could preserve the molecular diversity of prostate cancer subtypes.	(23)
Patient-derived benign prostate epithelial cells	Normal benign prostate tissue; Oncogenes (c-Myc + myrAKT1) introduced to induce malignant transformation	Matrigel; 20-30 μL Matrigel per culture well	Advanced DMEM/F12 (EGF, Noggin, R-spondin 1, A83-01, DHT, FGF2, FGF10, PGE2, nicotinamide, NAC, SB202190 (p38i), B27; Y-27632 added during seeding only)	NR	An organoid culture system enabling the propagation of basal cells and rare luminal progenitor cells was used to transform both populations of cells.	(24)
Patient-derived normal prostate tissues + genetically engineered mouse prostate epithelium	Human: normal benign prostate tissue; Mouse: normal prostate	Matrigel; NR	Human: Modified ENR medium; Mouse: ENR base medium (EGF, R-spondin1, Noggin, TGF-β/Alk inhibitor A83-01, DHT, FGF10, FGF2, PGE2, SB202190, and Nicotinamide)	NR	A 3D culture system that supports long-term expansion of primary mouse and human prostate organoids was described.	(20)
Patient-derived mCRPC biopsy samples	mCRPC	Matrigel; Cell suspension: Matrigel = 1: 2 (volume ratio), and 5-20 μL Matrigel-cell droplets plated per well	Advanced DMEM/F12 (EGF, Noggin, R-spondin 1, A83-01, DHT, FGF2, FGF10, PGE2, nicotinamide, NAC, SB202190, B27; Y-27632 supplemented in processing PBS)	NR	PDTO cultures were developed to screen the patients with metastatic prostate cancer for CHD1 loss.	(25)
Patient-derived metastatic prostate cancer biopsy samples + PDXs	mCRPC	Matrigel; NR	Advanced DMEM/F12 (B27, nicotinamide, NAC, EGF, Noggin, R-spondin-1, FGF2, FGF10, A83-01, SB202190, PGE2, DHT; Y-27632 only for tissue dissociation & seeding)	NR	PCOs could recapitulate the genotypic and phenotypic heterogeneity of metastatic prostate cancer, based on which relevant subtypes were validated.	(26)
Patient-derived radical prostatectomy samples	Treatment-naïve primary prostate adenocarcinoma	Matrigel; 20–000 cells resuspended in 40 μL 90% Matrigel per well	Advanced DMEM/F12 (B27, nicotinamide, NAC, EGF, A83-01, Noggin, R-spondin, DHT, FGF2, FGF10, PGE2, SB202190; Y-27632 added for first week only)	>90%	PCOs faithfully recapitulated the histological characteristics and lineage profiles of parental tumor tissues, while mimicking the heterogeneity.	(27)
Patient-derived radical prostatectomy samples	CRPC + hormone-naïve primary prostate adenocarcinoma	BME; Single cell suspension mixed with cold RGF BME, and 40 μL BME droplets per 24-well plate dome	Advanced DMEM/F12 (B27, nicotinamide, NAC, EGF, Noggin, R-spondin 1, FGF2, FGF10, DHT, A83-01, SB202190, PGE2; Y-27632 supplemented only during tissue digestion & passaging)	>60%	PCOs exhibited morphological resemblance to the corresponding primary tumors and displayed genetic clustering with prostate cancer.	(28)
Patient-derived samples from radical prostatectomy or transurethral resection of the prostate	Localized hormone-naïve prostate cancer + mCRPC	Matrigel; 50–000 tumor cells resuspended in 40 μL pure Matrigel per 24-well dome	Advanced DMEM/F12 (B27, nicotinamide, NAC, EGF, Noggin, R-spondin-1, FGF2, FGF10, A83-01, SB202190, PGE2; DHT: 1 nM for LPC, 0.1 nM for CRPC; Y-27632 added only on day 1 post-seeding)	100%	PCOs showed a high concordance in genomic landscape with parental tumors.	(29)
Patient-derived fresh metastatic prostate tumor biopsies/autopsy tumor tissues	CRPC-Adeno + CRPC-NEPC	Matrigel & synthetic PEG-4MAL hydrogel; Matrigel: Cell pellet mixed with Matrigel at 1:2 volume ratio, 50 μL droplets per well. PEG-4MAL: 20 mM PEG-4MAL stock, ligand final density 5×, crosslinker ratio VPM: DTT = 50:50, and cell suspension mixed 1:1 with crosslink solution	Advanced DMEM/F12 (B27, nicotinamide, NAC, EGF, Noggin, R-spondin-1, FGF2, FGF10, A83-01, SB202190, PGE2, DHT; Y-27632 supplemented only during tissue dissociation & seeding)	16%	Organoids maintained in synthetic hydrogel niches illustrated that ECM subtypes regulated the morphological, transcriptomic, and epigenetic hallmarks of CRPC-Adeno and CRPC-NEPC.	(30)

PDXs, patient-derived xenografts; PCOs, prostate cancer organoids; NR, not reported; CTCs, circulating tumor cells; CRPC, castration-resistant prostate cancer; BME, basement membrane extract; NEPC, neuroendocrine prostate cancer; ECM, extracellular matrix.

Unlike their 2D counterparts, organoids exhibit lower proliferation rates, which compromises their response to chemotherapeutic agents designed to target rapidly dividing cells (34, 35). Encapsulation-based culture approaches not only enable the generation of size-standardized cell aggregates but also can be combined with genomic and proteomic profiling techniques. These integrated strategies facilitate investigations into cellular growth kinetics, drug responses, and coculture assays with TME cells (30). Synthetic hydrogels are engineered to recapitulate the tumor-specific ECM environment through crosslinking ECM ligand polymers to form a 3D scaffold. When applied for the coculture of CRPC and neuroendocrine prostate cancer (NEPC) organoids, these hydrogels induce typical branching morphological phenotypes consistent with luminal cell loss during NEPC progression (30). Additionally, accumulating evidence demonstrates that well-characterized synthetic ECM platforms may provide more reliable drug sensitivity prediction results than Matrigel-based systems (36). Collectively, various scaffolds and culture systems have been established for PCO generation, with synthetic platforms overcoming Matrigel’s intrinsic drawbacks and offering promising alternative models for studying prostate cancer biology, drug response mechanisms and TME crosstalk.

## 4 PCOs for identification of cellular origin and heterogeneity recapitulation

The first breakthrough in establishing patient-derived PCO lines was reported in 2014. That study achieved long-term culture of 7 organoid lines derived from clinical biopsies and circulating tumor cells, and employed these models to investigate the initiation of prostate cancer (23). However, the organoids established from biopsies could only be maintained for 1–2 months, as the cultures were eventually overgrown by tumor-associated spindle cells or normal epithelial cells present in the original biopsy samples (23). Based on an R-spondin1-dependent culture system, it is found that both basal and luminal compartments harbor bipotent progenitors that can drive differentiation toward both lineages (20). Of note, although prostaspheres can likewise be serially passaged, those generated exclusively from basal-phenotype prostate stem cells are unable to undergo luminal differentiation under androgen stimulation (37). There is a study suggesting that prostate organoids can be established from luminal stem/progenitor populations referred to as castration-resistant Nkx3.1-expressing cells (CARNs), which retain functional AR signaling. Luminal cells exhibit a higher capacity for organoid formation and can give rise to basal cells in culture. Additionally, tumor organoids can arise from oncogenically transformed CARNs as well as from mouse models of prostate cancer, and can support the evaluation of drug responses (38). These findings highlight the progenitor characteristics of luminal cells and *in vitro* culture strategies of organoids for investigating prostate biology. When luminal-derived organoids were transduced with prostate cancer oncogenic drivers such as Myc and AKT1, they formed low-grade prostate adenocarcinomas. In contrast, introducing the same genetic alterations into basal cells led to the development of more aggressive tumors in mice, characterized by low/absent AR and prostate-specific antigen expression (24). Thus, identical oncogenic stimuli can result in distinct prostate cancer subtypes when targeting luminal and basal epithelial cells, respectively. Collectively, these studies advance our understanding of cellular origin and the properties of organoid-initiating cells in prostate cancer, and further validate PCOs as a reliable research platform for prostate cancer research.

In contrast to conventional 2D cultures, 3D cultures established from prostate cancer cell lines exhibit modified expression profiles of signaling molecules, phosphoproteins, and transcription factors, which contribute to their 3D growth characteristics. Furthermore, unique gene expression signatures have been recognized in organoids with distinct morphological phenotypes, underscoring the potential advantages of PDTOs in recapitulating both intra- and inter-tumor heterogeneity in prostate cancer (39). Importantly, PCOs also contribute to delineating the genomic landscape of advanced prostate cancer. Utilizing a 3D organoid system, Gao et al. demonstrated that the established organoid lines could reflect the molecular heterogeneity of prostate cancer subtypes, such as TMPRSS2-ERG fusion, SPOP mutation, SPINK1 overexpression, and CHD1 loss, while harboring a mutational profile similar to that of primary prostate cancer (23). Meanwhile, these organoid lines exhibited functional inactivation of p53 and RB tumor-suppressor pathways, a key driver alteration frequently observed in advanced prostate cancer. Importantly, the organoids could preserve genomic integrity for as long as six months compared with the original tumor genome.

PDTOs provide a versatile platform for introducing secondary genomic alterations via CRISPR/Cas9 and other gene-editing approaches. Lineage plasticity represents a key feature of aggressive prostate cancer, in which tumor cells lose AR dependence and progress to NEPC after AR-targeted therapy (8, 40). Elevated NSD2 expression in CRPC-neuroendocrine type (NE) was associated with unfavorable patient survival. In both PCOs generated from genetically engineered mice and human CRPC-NE organoids, CRISPR-mediated depletion of NSD2 was identified to reverse the CRPC-NE phenotype back to adenocarcinoma (41). Ladewig et al. updated the FOXA1 mutational profile in 874 primary and metastatic prostate cancer specimens. Based on a mouse prostate organoid model, they demonstrated that different types of FOXA1 mutations could drive cell differentiation toward two opposing cell fates: basal-like or luminal endocrine-like L1 subtypes (42). CDK12 inactivation showed variable co-occurrence with other hallmark mutations in prostate cancer, implying that its role in tumorigenesis was dependent on the accompanying mutational background (43). To identify mutations that synergize with or antagonize Cdk12 deficiency, CRISPR screening was performed in the organoid model, and Cdk12 deficiency was positively correlated with p53 inactivation positively (44). Moreover, CRISPR screening based on PDTOs utilized in the study of Venkadakrishnan et al. effectively captured the lineage transition, and the key transcription factor PROX1 was identified (45). Collectively, patient-derived and genetically engineered organoids enable accurate functional validation of genomic alterations, cell fate transitions, and genotype-phenotype relationships in advanced and therapy-resistant prostate cancer.

## 5 PCOs for modeling TME interactions

Prostate cancer progression is tightly modulated by the TME, which drives tumor dissemination and reshapes therapeutic responses to anticancer treatments (46). Multiple studies have established co-culture systems of prostate cancer cells with non-immune stromal subsets, including cancer-associated fibroblasts (CAFs) and endothelial cells, to simulate TME crosstalk (39, 47). Metastatic prostate cancer exhibits prominent bone tropism, and osteoblastic lesion formation reflects the strict dependence of metastatic tumor cells on the bone microenvironment for disease progression. In a defined 3D hydrogel model that could support the co-culture of prostate cancer PDX cells and osteoblastic cells, the prostate cancer-osteoblast unit was simulated successfully within the bone metastatic environment *in vitro* (47). Furthermore, mesenchymal stem cells are also the predominant infiltrating cell type in prostate cancer, and facilitate tumor progression via the induction of tumor-promoting CAFs. The co-culture of prostate cancer cells with CAFs has been widely utilized to recapitulate TME interactions and explore targeted therapeutic strategies (39). Notably, activated CAFs exert a profound effect on the drug response of tumor cells, although they are ultimately outcompeted by tumor cell proliferation (48). Co-culturing prostate cancer cells with CAFs also upregulates cholesterol metabolism and steroid biosynthesis in prostate cancer cells, thereby inducing resistance to AR-targeted therapy (49).

Recently, PCOs have been co-cultured with primary prostate stromal cells, including fibroblasts and smooth muscle cells. Richards et al. established a human prostate organoid co-culture system incorporating prostate stromal cells, and demonstrated that the organoids from prostate cancer lesions preserved differential expression of alpha-methylacyl-CoA racemase. Moreover, this co-culture also enhanced the organoid viability and enabled extended passaging (50), underscoring the ability of PCOs to better recapitulate native tissue characteristics when stromal components are combined with epithelial cells. In another study, 2D and 3D mono- and co-cultures of prostate cancer cells and CAFs were applied to mimic tumor-stroma interactions within the ECM, and CAFs showed a marked protective effect on FGFR inhibitor treatment in both 2D and 3D co-cultures (51). Lee et al. generated prostate cancer assembloids from localized prostate cancer and CRPC tissues by combining tumor organoids with patient-matched CAFs to reconstruct the TME. These assembloids maintained high genomic consistency to parental tumors, better mimicked primary tumor phenotypes, and displayed enhanced proliferation and drug resistance, validating their utility for studying tumor-stromal crosstalk and personalized therapy (29). Furthermore, Monteiro et al. demonstrated that incorporating stromal components was essential for establishing stable, morphologically uniform prostate cancer spheroids. Compared with monotypic spheroids, the optimized heterotypic models showed higher treatment resistance and were more suitable for drug screening (52). Together, these findings highlight that stroma-integrated PCOs represent a physiologically relevant and powerful alternative model for better simulating prostate cancer pathophysiology, dissecting tumor-stroma crosstalk, and advancing personalized treatment discovery.

## 6 PCOs for drug sensitivity testing

The emergence of PDTOs has greatly improved the reliability of drug screening for prostate cancer. Compared with traditional 2D cell cultures, PDTOs can better recapitulate the architectural characteristics and native microenvironmental traits of parental tumors, thereby conferring higher physiological relevance for drug efficacy evaluation. In recent years, CRPC-derived organoids have been employed to assess the therapeutic efficacy of enzalutamide, everolimus, and BKM-120 (53–55). It has been reported that the MSK-PCa2 organoid line shows high sensitivity to enzalutamide, whereas other prostate cancer cell line-derived organoids display inherent resistant. Compared with wild-type MSK-PCa2 organoids, those harboring *PTEN* deletion and *PIK3R1* mutations exhibit increased sensitivity to everolimus and BKM-120 (23). Thus, these data support the potential of PCOs as a feasible platform for mechanistic research and drug screening.

In previous studies, 4 CRPC-NE organoids and 2 CRPC-adenocarcinoma organoids were generated for high-throughput screening of 129 chemotherapeutic and targeted agents (16, 56). These studies demonstrated superior efficacy of AR antagonists like enzalutamide and chemotherapeutic drugs, such as docetaxel and cabazitaxel, in CRPC-derived organoids. Additionally, investigational agents, such as pozotinib and vandetanib, had also been evaluated in PCOs, both of which exerted potent inhibitory effects specifically against CRPC-NE organoids (16). In another study, Jansson et al. conducted high-throughput screening of more than 100 compounds using CRPC LuCaP PDX-derived organoids, among which HSP90 inhibitors displayed marked antitumor activity (57). Notably, ganetespib, one of the HSP90 inhibitors, showed the most potent inhibitory effects in CRPC LuCaP PDX models mainly through simultaneous targeting of the AR and PI3K signaling pathways (57). Recently, Qian et al. generated PCOs from patient biopsy samples, and drug screening demonstrated heterogeneous responses of these PCOs to enzalutamide, docetaxel, and olaparib, supporting the potential of PCOs as a promising preclinical model for treatment response prediction (58). Moreover, the optimized heterotypic spheroids were also utilized for standard-of-care drug screening, showing greater treatment resistance relative to monotypic spheroids (52).

## 7 PCOs for studying the mechanisms of drug resistance

Drug resistance in tumor cells is primarily driven by genetic mutations, chromosomal amplification, and structural rearrangements. In CRPC, there are three key mechanisms proposed to underlie drug resistance: (1) alterations in *AR*, *ETS*, *TP53*, *PTEN* genes and in AR and PI3K signaling pathways; (2) abnormal RNA splicing that can lead to a signaling switch from AR to the glucocorticoid receptor pathway; and (3) acquired resistance arising from enhanced lineage plasticity following prolonged drug exposure (59–61).

PCOs have emerged as a valuable 3D model for dissecting the molecular mechanisms underlying drug resistance in prostate cancer (62). In a previous study, PCOs were used as a model system to investigate the efficacy of anti-androgen agents against p53- and PTEN-deficient prostate cancer (63). Furthermore, emerging evidence has demonstrated that *SPOP* mutations contribute to the development of resistance to BET inhibitors based on PCOs (64, 65). Importantly, drug-resistant PCOs can also be derived from patients with clinically acquired resistance to anticancer agents, or established *in vitro* through sustained exposure to anticancer drugs to induce a resistant phenotype (66). A notable example is the generation of androgen deprivation therapy-resistant PCOs by culturing cells under androgen-depleted conditions or in the presence of antiandrogens, such as enzalutamide (67).

Through organoid models, xenograft models, and clinical patient samples, Dhimolea et al. explored treatment-resistant tumor cells, and found that inhibition of BRD4, a key co-activator of MYC, enhanced drug resistance while decreasing the drug-induced cytotoxicity (68). By integrating patient tumor proteomics, RNA sequencing, spatial omics, and synthetic hydrogel-based organoids, treatment resistance in CRPC-NE was found to be reversed under ECM-related drug-resistant conditions via sequential therapy: initial cellular reprogramming using epigenetic inhibitors, followed by DRD2 targeting (30). These synthetic organoid models highlight the regulatory function of the ECM in modulating treatment responses to targeted therapy in CRPC-NE and facilitate the identification of novel strategies to overcome drug resistance. Additionally, aberrant JAK-STAT activation can drive lineage plasticity and stem-like properties in PCOs, leading to resistance against AR-targeted therapy, whereas JAK-STAT inhibition restores drug sensitivity (69). Notably, PCOs faithfully recapitulate the heterogeneity of CRPC-NE and the process of NE differentiation, and NSD2 inhibition effectively reverses drug resistance mediated by tumor cell plasticity in CRPC (41). Overall, as a promising preclinical model, PCOs enable systematic assessment of drug responses and mechanistic investigation into the development of drug resistance in prostate cancer.

## 8 PCOs for precision medicine

Over the past few years, more and more evidence has supported the utility of PCOs in advancing precision medicine for prostate cancer. In *BRCA2*^-/-^ PCOs, the PARP inhibitor olaparib showed the strongest cytotoxic effect, consistent with clinical patient responses (15). This enhanced sensitivity of CRPC patients to olaparib was further functionally confirmed in another study based on CRPC organoids (28). Importantly, in a phase II trial of the aurora kinase A inhibitor alisertib for CRPC-NE, PCOs established from pretreatment biopsies displayed consistent treatment responses with clinical observations, enabling dynamic assessment of Aurora-N-myc complex disruption (70). Qian et al. generated PCOs from targeted biopsies, and found that these PCOs exhibited significant heterogeneity while maintaining good response to enzalutamide, docetaxel, and olaparib, confirming their potential as a preclinical model for treatment response prediction (58). Moreover, to enhance the success rate and lower the cost of generating advanced PCOs from transurethral resection of prostate specimens, Ali et al. optimized the organoid culture protocol and medium. They demonstrated that the established PCOs could largely retain the key molecular and pathological features of the corresponding primary tumor tissues (71). Moreover, the drug sensitivity results derived from these PCOs showed a significant correlation with patient clinical outcomes (71). Collectively, these PCO models have the potential to assist in accurate prediction of patient treatment responses, thereby supporting clinical treatment decision-making.

## 9 Challenges

Organoid technology has advanced significantly in prostate cancer research, particularly in TME modeling, drug screening, and mechanistic investigation of drug resistance. A diverse range of naturally occurring genetic mutations is present in CRPC organoids, enabling these organoids to act as new platforms for testing mechanistic hypotheses and facilitating discovery through molecular analyses, including gene-based screening and responses to environmental manipulation. With continuous technological development, organoids are expected to play an increasingly pivotal role in the clinical translation of prostate cancer research, thereby promoting the achievement of early diagnosis, individualized treatment, and precision medicine. Nevertheless, this technology still confronts several major challenges that need to be addressed for further advancement.

First, organoid culture is highly dependent on specific culture conditions. Precise control of nutritional components, growth factors, and matrix components is required to ensure that organoids better simulate the 3D structure and physiological function of native tissues. In addition, organoid culture relies on complex technical support and high-precision equipment, placing higher demands on researchers’ technical skills and laboratory resources. A key area of focus to address this challenge lies in the establishment of refined culture systems that support prostate epithelial cells alongside stromal and immune components of the normal or TME. A reductionist strategy in co-culture systems permits dissection of how defined TME components modulate the growth of prostate normal or tumor-derived organoids. For instance, stromal- and immune-derived NRG1 has been shown to sustain the survival of prostate luminal progenitor cells under androgen signaling inhibition, thereby driving CRPC progression, which have been validated in organoid and xenograft models (72). Therefore, optimizing the organoid culture system and improving the stability and consistency of the culture process, including the integration of TME components, remain a key issue in current research.

Second, prostate cancer exhibits significant heterogeneity, posing enormous challenges to clinical treatment. A critical yet frequently underappreciated limitation of PCO cultures is that benign-like epithelial populations can progressively outgrow malignant cells during in-vitro expansion (73). Consequently, tumor clones bearing prostate cancer molecular hallmarks tend to be lost upon serial passaging, even when malignant cells are readily detectable in early-passage cultures. Additionally, PCO cultures suffer from intrinsic in-vitro selection pressure, whereby predominantly aggressive, advanced, and NE-like tumor subpopulations survive and expand. This limitation restricts their capacity to faithfully recapitulate the full pathological diversity of clinical prostate cancer (74). Although organoid models can largely recapitulate the biological characteristics of individual tumors, there are still significant differences in morphology, proliferative rates, and drug sensitivity among organoids from different sources, limiting their performance in reproducibility and universality. Prostatic stroma is recognized as a key mediator of responses to androgen deprivation therapy, implying the presence of androgen-responsive growth-promoting cells within the prostate microenvironment (75). Similarly, the consistent bone-homing tendency of metastatic prostate cancer indicates the existence of growth-supportive niches in the bone marrow (76, 77). Therefore, identification and incorporation of such growth-supportive cell types into organoid coculture systems, together with further optimization of culture media and ECM components, constitute promising strategies to address these challenges, including efforts to reduce culture-driven selection and preserve broader subclonal diversity within organoid preparations.

Third, the existing organoid models still struggle to fully recapitulate the complex TME of prostate cancer, which is composed of multiple components including diverse cell types, vascular systems, immune components, and exosomes. The crosstalk among these elements plays a decisive role in tumor progression, metastasis, and treatment response. Most currently available organoid models rely on relatively simplified matrix supports and only contain a limited number of cell types, which greatly restricts their ability to reproduce the complex interactions within the native microenvironment, such as the crosstalk between CAFs, immune cells, and endothelial cells. This limitation is particularly relevant in immuno-oncology, an area where organoid models have proven highly useful for both the evaluation and potential discovery of immuno-oncology therapeutics (78). For example, PDTOs can be co-cultured with autologous patient peripheral blood lymphocytes to assess the cytotoxic activity in response to immuno-oncology drugs, while enabling the amplification and identification of tumor-reactive T cell receptors (79, 80). To improve the organoid physiological relevance, several recent studies have focused on applying bioengineering technologies to integrate dynamic factors, such as vascular networks, immune cells, and CAFs, into them (29, 81, 82). In this way, organoids can more closely simulate the interactions between tumors and their surrounding microenvironment, thereby providing a relatively reliable experimental platform for drug screening, development of immunotherapeutic regimens, and study of drug resistance mechanisms.

## 10 Conclusions

PCOs represent a promising alternative preclinical model that effectively overcomes the limitations of conventional cell lines and PDXs. PCOs can largely recapitulate the genomic heterogeneity, lineage plasticity, TME crosstalk, and drug response profiles of primary and metastatic prostate cancer, enabling mechanistic studies of tumor initiation, progression, and treatment resistance. They serve as reliable platforms for high-throughput drug screening, genotype-phenotype validation, and precision medicine, supporting individualized therapeutic selection and biomarker identification. However, challenges remain, including low establishment efficiency, high dependence on specialized culture conditions, inter-sample variability, and incomplete recapitulation of the complex TME. Future advances in synthetic matrices, multicellular coculture systems, and bioengineering will further enhance the physiological relevance and reproducibility of PCO models. With continued optimization, PCOs will potentially accelerate translational research and clinical implementation, ultimately improving diagnosis, treatment, and prognosis for prostate cancer patients.
